# DiCoDi® (Digital Cognitive Diagnostics): Ein tabletbasierter kognitiver Test für Menschen mit und ohne Schwerhörigkeit

**DOI:** 10.1007/s00106-026-01722-8

**Published:** 2026-02-02

**Authors:** I. Ballasch, M. Meka, E. Kalbe, J.-P. Schmid, C. Vorstius, A. Koj, J. Kessler

**Affiliations:** 1 Klinik und Poliklinik für Neurologie, Uniklinik Köln, Köln, Deutschland; 2 Medizinische Psychologie | Neuropsychologie und Gender Studies & Centrum für Neuropsychologische Diagnostik und Intervention (CeNDI), Medizinische Fakultät und Uniklinik Köln, Universität zu Köln, Köln, Deutschland; 3 KOJ hearing network, Achern, Deutschland; 4 Institut für Psychologie, Bergische Universität Wuppertal, Wuppertal, Deutschland

**Keywords:** Demenz, Mittleres Lebensalter, Hörvermögen, Früherkennung, Kognitive Beeinträchtigung, Dementia, Midlife, Hearing ability, Early detection, Cognitive impairment

## Abstract

**Hintergrund:**

Eine Schwerhörigkeit im mittleren Lebensalter gilt als einer der wichtigsten modifizierbaren Risikofaktoren für eine Demenz. Bei der Durchführung herkömmlicher kognitiver Tests werden Beeinträchtigungen des Hörvermögens allerdings meist nicht berücksichtigt.

**Ziel der Arbeit:**

Ziel waren die Vorstellung und Evaluierung des DiCoDi® (Digital Cognitive Diagnostics), eines digitalen kognitiven Tests für Menschen mit und ohne Schwerhörigkeit.

**Material und Methoden:**

Der DiCoDi® umfasst 9 Subtests, ist hörunabhängig, selbstständig durchführbar und dauert 20–30 min. Es wurden 373 Personen untersucht: 204 Kontrollpersonen (Alter: 67,48 Jahre [12,2], Frauen: 58,0 %) und 173 Personen mit Schwerhörigkeit (Alter: 72,86 Jahre [8,7], Frauen: 46,8 %). Basierend auf einem kombinierten kognitiven Index wurden 2 Gruppen gebildet: 314 Personen ohne (Alter: 69,38 Jahre [12,2], Frauen: 54,1 %) und 58 Personen mit kognitiver Beeinträchtigung (Alter: 72,93 Jahre [12,2], Frauen: 46,6 %). Zusätzlich kam eine umfassende neuropsychologische Testbatterie zum Einsatz.

**Ergebnisse:**

In den meisten DiCoDi®-Subtests unterschieden sich Kontrollpersonen und Personen mit Schwerhörigkeit nicht signifikant. Personen mit Schwerhörigkeit erzielten in den Subtests „Trail Making A“ (_p_η^2^ = 0,24) und „Zeitgefühl“ (_p_η^2^ = 0,05) signifikant bessere Leistungen als die Kontrollpersonen. Personen mit kognitiver Beeinträchtigung schnitten in den meisten DiCoDi®-Subtests signifikant schlechter ab (0,13 ≤ _p_η^2^ ≤ 0,28). Der DiCoDi® zeigte eine hohe Validität, Reliabilität und gute Klassifikationsgenauigkeit (AUC, „area under the curve“ = 0,80).

**Diskussion:**

Die Ergebnisse zeigen, dass der DiCoDi® ein valides und reliables Instrument zur Früherkennung kognitiver Defizite bei Menschen mit und ohne Schwerhörigkeit sein kann.

**Zusatzmaterial online:**

Die Online-Version dieses Beitrags (https://doi.org/10.1007/s00106-026-01722-8) enthält die Tabellen S1–S5.

## Abstract (en)

**Background:**

Hearing loss in midlife is considered one of the most important modifiable risk factors for dementia; however, standard cognitive tests often fail to adequately detect cognitive deficits in individuals with hearing loss.

**Objective:**

The aim of the study was to present and evaluate the digital cognitive diagnostics (DiCoDi®), a digital cognitive test designed for individuals with and without hearing loss.

**Material and methods:**

The DiCoDi® comprises 9 subtests, is independent of hearing, can be self-administered and takes 20–30 min to complete. A total of 373 individuals were assessed: 204 control participants (mean age: 67.48±12.2 years, women: 58.0 %) and 173 individuals with hearing loss (mean age: 72.86±8.7 years, women: 46.8 %). Based on a combined cognitive index 2 groups were formed: 314 individuals without cognitive impairment (age: 69.38±12.2 years, women: 54.1 %) and 58 individuals with cognitive impairment (age: 72.93±12.2 years, women: 46.6 %). In addition, a comprehensive neuropsychological test battery was employed.

**Results:**

In most DiCoDi® subtests there were no significant differences between control participants and individuals with hearing loss. Participants with hearing loss performed significantly better than controls in the subtests “Trail Making A” (_p_η^2^ = 0.24) and “Time Estimation” (_p_η^2^ = 0.05). Individuals with cognitive impairment performed significantly worse in most DiCoDi® subtests (0.13 ≤ _p_η^2^ ≤ 0.28). The DiCoDi® demonstrated high validity, high reliability and good classification accuracy (area under the curve, AUC = 0.80).

**Conclusion:**

The results show that the DiCoDi® can be a valid and reliable tool for the early detection of cognitive deficits in people with and without hearing loss.

## Kurze Hinführung zum Thema

Die steigende Zahl älterer Menschen mit sowohl auditiven als auch kognitiven Beeinträchtigungen unterstreicht die dringende Notwendigkeit, einen kognitiven Test zu entwickeln, der Schwerhörigkeit berücksichtigt und gleichzeitig im klinischen Alltag anwendbar ist.

## Hintergrund und Fragestellung

Einschränkungen des Hörvermögens gehen mit einem erhöhten Risiko für kognitive Defizite und einer Demenz einher [1]. Livingston et al. [2] identifizierten eine Schwerhörigkeit im mittleren Lebensalter als eines der wichtigsten modifizierbaren Risiken einer Demenz. Personen mit Schwerhörigkeit werden allerdings von etablierten kognitiven Tests wie dem Montreal Cognitive Assessment (MoCA; [3]), Demenz-Detektions-Test (DemTect; [4]) und Mini-Mental-Status-Test (MMST; [5]) nur unzureichend erfasst, ihre Leistungen unterschätzt oder sogar falsche Diagnosen (falsch-positiv) gestellt, da diese Verfahren von einem intakten Sensorium ausgehen [6].

Einzelne Anpassungen bestehender kognitiver Tests für Personen mit Schwerhörigkeit existieren, darunter Hearing-Impaired MoCA (HI-MoCA; [7]), MoCA for People with Hearing Impairment (MoCA‑H; [8]), MMST für Hörgeminderte [9], O‑DEM [10] und DemTect^Ear^ [11]. Aufgrund der begrenzten Sensitivität und des Mangels an Validierungsstudien sowie deutschen Normdaten gelten aktuell nur MoCA‑H und O‑DEM als geeignete kognitive Screeningverfahren für Menschen mit Schwerhörigkeit. Die Tests werden vorwiegend in neurologischen oder geriatrischen Praxen eingesetzt, da ihre Komplexität Zeit und fundierte Kenntnisse in Durchführung und Auswertung erfordern. Ihre Paper-and-pencil-Basis verursacht zudem Material‑, Zeit- und Personalkosten.

Zur Überwindung dieser Einschränkungen eignen sich die tabletgestützte Cambridge Neuropsychological Test Automated Battery (CANTAB; [12]) und die computergestützte ALAcog (ALA: Institut für Arbeiten Lernen Altern/cog: Kognition)-Testbatterie [13]. Beide sind jedoch aufgrund langer Testdauer, erforderlicher Fachkenntnisse und notwendiger spezieller Schulungen im klinischen Alltag nur eingeschränkt einsetzbar [14].

Vor diesem Hintergrund wurde der DiCoDi® (Digital Cognitive Diagnostics) entwickelt – ein tabletbasierter kognitiver Test für Personen mit und ohne Schwerhörigkeit. In der vorliegenden Studie wurde untersucht, ob Unterschiede im DiCoDi® zwischen Menschen mit und ohne Schwerhörigkeit vorliegen. Zudem wurde der DiCoDi® hinsichtlich seiner Validität und Reliabilität evaluiert. Darüber hinaus wurde untersucht, ob und wie sensitiv der DiCoDi® zwischen altersgerechten kognitiven Leistungen und kognitiver Beeinträchtigung differenzieren kann.

## Studiendesign und Untersuchungsmethoden

### DiCoDi®

**Abb. 1:**
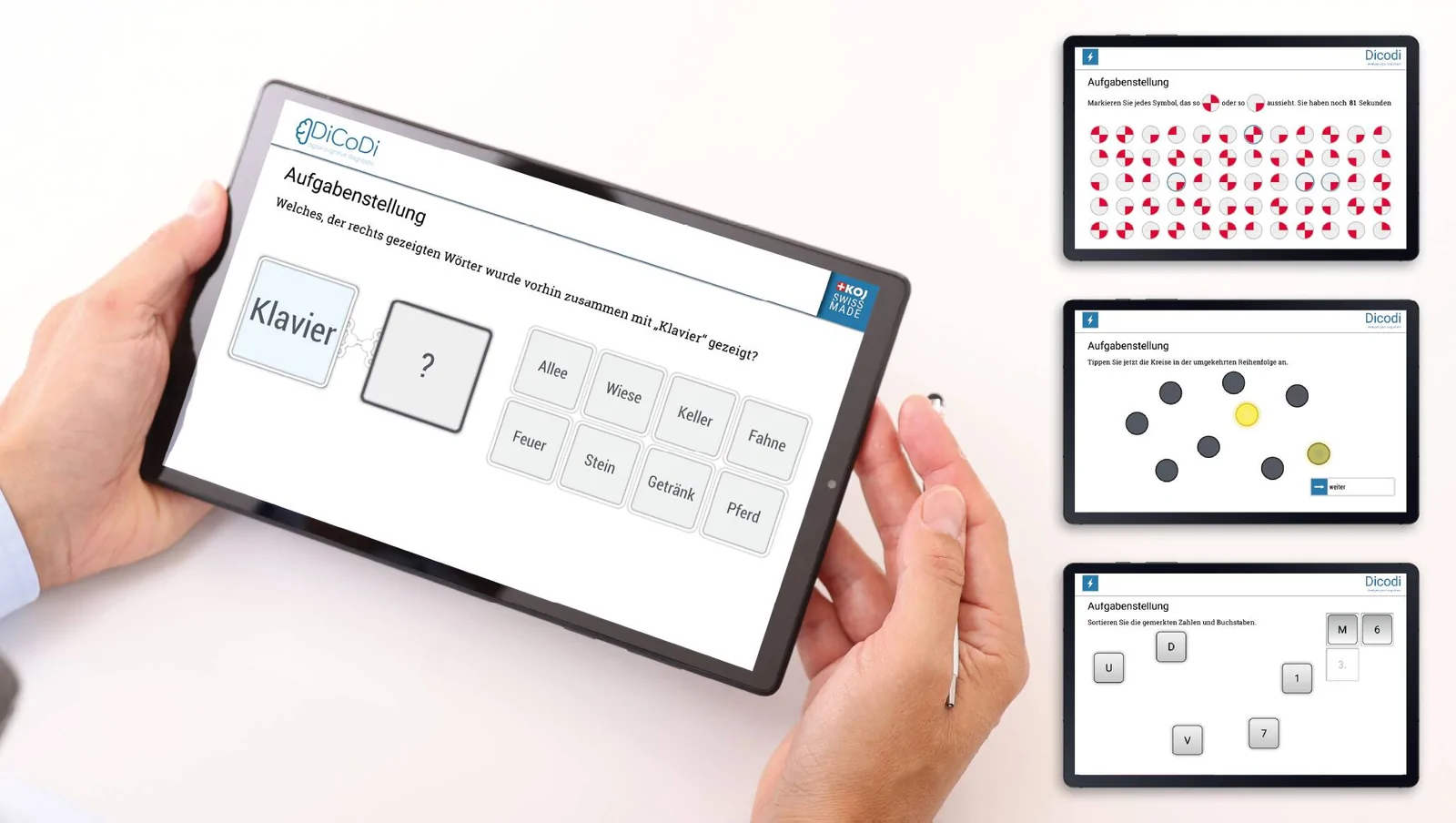
DiCoDi®(Digital Cognitive Diagnostics)-Subtests: Wortpaare merken (*links*), Konzentration und Aufmerksamkeit (*oben rechts*), Kreishüpfen (*mittig rechts*) sowie Zahlen und Buchstaben sortieren (*unten rechts*)

Der DiCoDi® ist ein digitaler kognitiver Test für Menschen mit und ohne Schwerhörigkeit ab 40 Jahren, da eine Schwerhörigkeit typischerweise ab dem 40. Lebensjahr beginnt [15]. Er besteht aus 9 Subtests (siehe Abb. 1) sowie einer Selbsteinschätzung zu kognitiven und affektiven Funktionen auf einer 4‑stufigen Likert-Skala. Tabelle S1 im Zusatzmaterial online liefert eine Übersicht über die DiCoDi®-Subtests. Alle Subtests wurden auf Basis etablierter kognitiver Tests (siehe Tab. S1) entwickelt und für die Durchführung auf dem Tablet entsprechend modifiziert. Der DiCoDi® dauert 20–30 min. Die Items und Instruktionen werden visuell auf einem Tablet dargeboten (Mindestschriftgröße: 12). Der DiCoDi® kann unter Aufsicht einer Testleitung selbstständig durchgeführt werden. Die Auswertung erfolgt computerbasiert. Der DiCoDi® ist bei KOJ hearing network (Achern, Deutschland; www.dicodi.de) für qualifizierte Berufsgruppen erhältlich.

### Stichprobenbeschreibung

Es wurden 373 Personen im Zeitraum von 2022 bis 2023 in der vorliegenden Querschnittsstudie untersucht, davon 200 Personen in der normalhörenden Kontrollgruppe (KG) und 173 Personen mit Schwerhörigkeit (nach World Health Organization [WHO]; [16]: leicht: *n* = 93 [53,9 %], mittel: *n* = 77 [44,5 %], schwer: *n* = 2 [1,2 %], an Taubheit grenzende Schwerhörigkeit oder Taubheit: *n* = 1 [0,6 %]). Der durchschnittliche Hörschwellenwert der Personen mit Schwerhörigkeit betrug 44,06 dB (11,6) für das rechte Ohr, 42,89 dB (10,6) für das linke Ohr und 43,27 dB (10,2) über beide Ohren gemittelt. Tab. 1 zeigt die soziodemographischen Charakteristika der Stichproben.

**Tab. 1:** Soziodemographische Zusammensetzung der Stichproben

	Alter in Jahren: *M* (*SD*) [Range]	Geschlecht ♀:♂: *n* (%)	Bildung in Jahren: *M* (*SD*) [Range]
Kontrollgruppe (*n* = 200)	67,48 (12,2) [50,0–89,0]	116 (58,0 %):84 (42,0 %)	13,57 (3,0) [8,0–23,0]
Personen mit Schwerhörigkeit (*n* = 173)	72,86 (8,7) [43,0–87,0]	81 (46,8 %):92 (53,2 %)	14,10 (2,4) [6,0–19,0]
Re-Test-Reliabilität Stichprobe (*n *= 71)	59,86 (9,5) [49,0–86,0]	40 (56,3 %):31 (43,7 %)	13,66 (2,4) [8,0–19,0]

Bildung in Jahren = Summe der Schulbildung und möglicher weiterführender Bildung wie Berufslehre/Studium, *M* Mittelwert, *SD* „standard deviation“

**Abb. 2:**
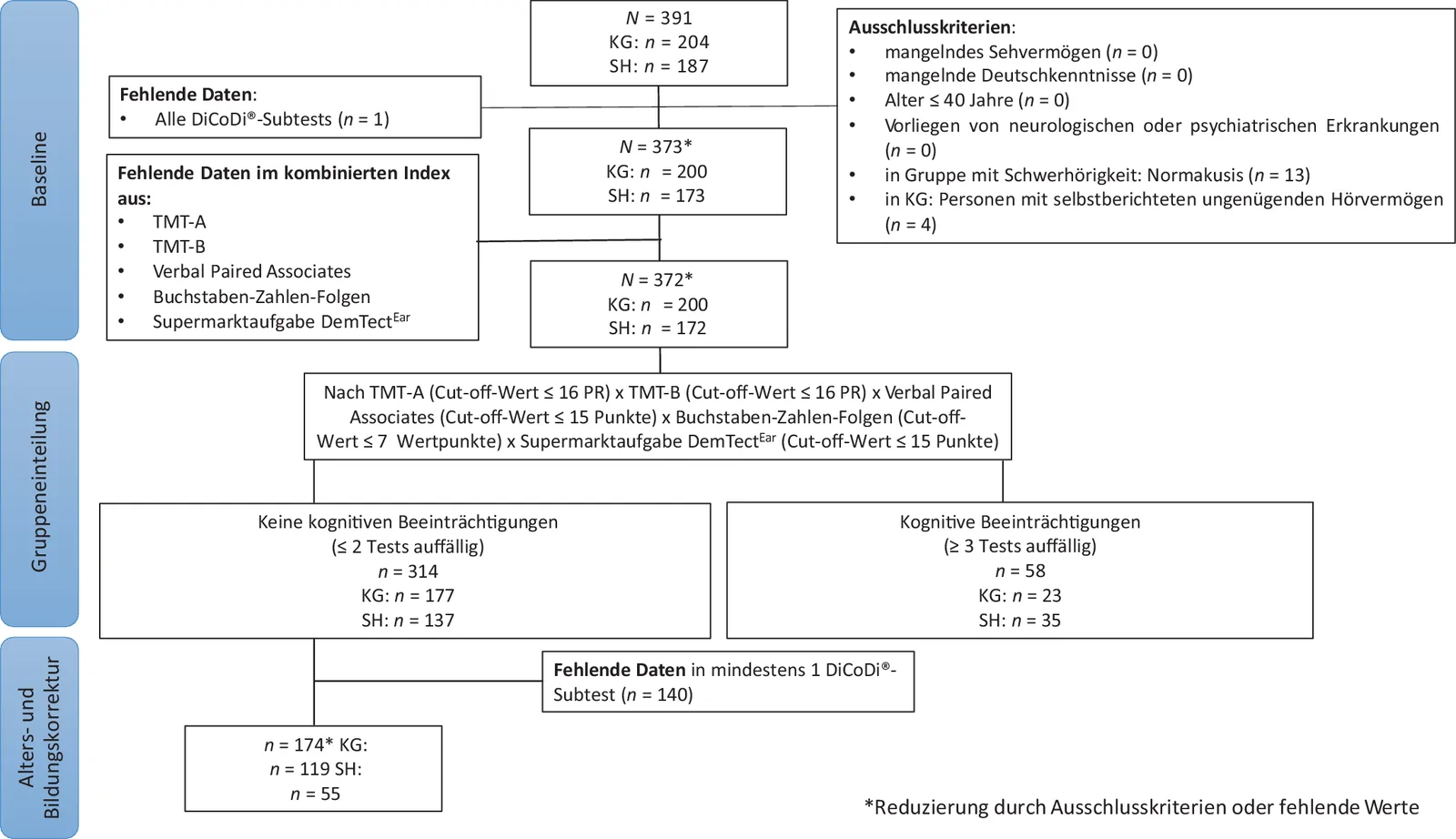
Übersicht der Stichproben (*DemTect*^*Ear*^ Demenz-Detektions-Test Ohr, *DiCoDi*® Digital Cognitive Diagnostics, *KG* Kontrollgruppe, *PR* Prozentrang, *SH* Personen mit Schwerhörigkeit, *TMT* Trail-Making-Test)

Menschen mit neurologischen oder psychiatrischen Erkrankungen und fehlenden Daten in allen DiCoDi®-Subtests wurden ausgeschlossen (*n* = 1). In der Gruppe mit Schwerhörigkeit führte das Vorliegen eines normalen Hörvermögens (Normakusis) zum Ausschluss (*n* = 13), während in der KG Personen mit selbstberichtetem ungenügenden Hörvermögen ausgeschlossen wurden (*n* = 4). Die Einschlusskriterien für beide Gruppen umfassten ein ausreichendes oder ausreichend korrigiertes Sehvermögen, ausreichende Deutschkenntnisse und ein Mindestalter von 40 Jahren (siehe Abb. 2).

Zusätzlich wurde eine Stichprobe aus 71 normalhörenden Kontrollpersonen zu 2 Testzeitpunkten zur Untersuchung der Re-Test-Reliabilität im Zeitraum von 2023 bis 2024 entsprechend den Bedingungen der KG rekrutiert und untersucht (siehe Abb. 2).

### Rekrutierung und Durchführung der Untersuchung

Die Rekrutierung der KG erfolgte über geschulte Medizin- und Psychologiestudierende im Großraum Köln. Die Personen mit Schwerhörigkeit wurden in den Akustikerfilialen der Firma KOJ in der Schweiz durch geschulte Psychologiestudierende getestet. Neben einem soziodemographischen Fragebogen und dem DiCoDi® wurde eine ausführliche neuropsychologische Testbatterie, bestehend aus den Paper-and-pencil-Tests für die Validierung (siehe Tab. S1 und S2) sowie dem DemTect^Ear^ [11], dem Beck-Depressions-Inventar‑V (BDI‑V; [17]) und dem Fragebogen „Kölner Ausgangsvoraussetzungen für neuropsychologische Testungen“ (KANT; [18]), durchgeführt. Zusätzlich wurde bei den Personen mit Schwerhörigkeit ein seitengetrenntes Reintonaudiogramm in den Frequenzen von 250 bis 8000 Hertz durchgeführt. Beeinträchtigungen des Hörvermögens in der KG wurden anhand anamnestischer Angaben und der Einschätzung der Testleitung erfragt. Zur Erfassung des Hörvermögens diente eine 4‑stufige Skala (sehr gut, gut, mittelmäßig, ungenügend). Nach Einschätzung der Testleitung waren alle Teilnehmenden in der Lage, in Zimmerlautstärke zu kommunizieren.

Alle beschriebenen Untersuchungen am Menschen wurden mit Zustimmung der Ethikkommission der Bergischen Universität Wuppertal (Aktenzeichen: MS/AE 220614) im Einklang mit nationalem Recht und gemäß der Deklaration von Helsinki von 1975 durchgeführt.

Anhand eines kombinierten Index, bestehend aus Trail-Making-Test A (TMT‑A; [19]; Cut-off-Wert ≤ 16 Prozentrang [PR]), Trail-Making-Test B (TMT‑B; [19]; Cut-off-Wert ≤ 16 PR), Verbal Paired Associates der Wechsler Memory Scale-IV (WMS-IV; [20]; Cut-off-Wert ≤ 15 Punkte), Buchstaben-Zahlen-Folgen der Wechsler Adult Intelligence Scale (WAIS; [21]; Cut-off-Wert ≤ 7 Wertpunkte) und der Supermarktaufgabe des DemTect^Ear^ ([11]; Cut-off-Wert ≤ 15 Punkte), wurde die gemeinsame Stichprobe aus KG und Personen mit Schwerhörigkeit in 2 Gruppen eingeteilt (siehe Abb. 2):

- ohne kognitive Beeinträchtigung (*n* = 314; 84,4 %),
- mit kognitiver Beeinträchtigung (*n* = 58; 15,6 %).

Die Cut-off-Werte entsprechen dem Mittelwert (*M*) abzüglich einer Standardabweichung für alle Tests, mit Ausnahme der Supermarktaufgabe des DemTect^Ear^. Aufgrund der hohen Streuung wurde hier ein Cut-off-Wert von weniger als 1 Standardabweichung verwendet (*M* = 20,07 [7,2]). Für die Einteilung in die Gruppe mit kognitiver Beeinträchtigung mussten auffällige Ergebnisse in mindestens 3 der genannten Tests vorliegen. Die 314 Personen ohne kognitive Beeinträchtigung (KG: *n* = 177; Schwerhörigkeit: *n* = 137) bestanden aus 170 Frauen (54,1 %), hatten ein Durchschnittsalter von 69,38 (11,1) Jahren und eine mittlere Schulbildungsdauer von 13,99 (2,7) Jahren. Die 58 Personen mit kognitiver Beeinträchtigung (KG: *n* = 23; Schwerhörigkeit: *n* = 35) umfassten 27 Frauen (46,6 %), waren im Mittel 72,93 (9,8) Jahre alt und wiesen durchschnittlich 12,95 (2,7) Schulbildungsjahre auf.

### Statistische Analysen

Die statistischen Analysen wurden mit SPSS Statistics 29.0 durchgeführt. Das Signifikanzniveau wurde auf α = 0,05 festgesetzt.

#### Gruppenunterschiede: KG vs. Schwerhörigkeit

Um Gruppenunterschiede (KG vs. Personen mit Schwerhörigkeit) in den DiCoDi®-Subtests zu ermitteln, wurden Kovarianzanalysen („analysis of covariance“, ANCOVA) mit der Gruppe als unabhängige Variable, Alter und Bildung als Kovariaten sowie den DiCoDi®-Subtests als abhängige Variablen berechnet. Das Signifikanzniveau wurde mithilfe der Bonferroni-Korrektur auf α = 0,0055 pro Test (0,05/9) korrigiert.

#### Gruppenunterschiede: Personen mit vs. ohne kognitive Beeinträchtigung

Um Gruppenunterschiede (Personen mit vs. ohne kognitive Beeinträchtigung) in den DiCoDi®-Subtests zu ermitteln, wurden ANCOVA mit dem kognitiven Status (mit vs. ohne Beeinträchtigung) als unabhängige Variable, Alter und Bildung als Kovariaten sowie den DiCoDi®-Subtests als abhängige Variablen berechnet. Das Signifikanzniveau wurde mithilfe der Bonferroni-Korrektur auf α = 0,0055 pro Test (0,05/9) korrigiert.

#### Konvergente und divergente Validität

Zur Bestimmung der konvergenten und divergenten Validität des DiCoDi® wurden 2‑seitige Spearman-Rangkorrelationen mit den dazugehörigen Paper-and-pencil-Tests der DiCoDi®-Subtests und dem inhaltsfremden Verfahren BDI‑V berechnet. Da sich die KG und Personen mit einer Schwerhörigkeit in den DiCoDi®-Subtests weitestgehend nicht unterschieden, wurden die Analysen in der gemeinsamen Stichprobe durchgeführt. Es wurde die Bonferroni-Korrektur angewandt (konvergente Validität: α = 000694 [0,05/72]; divergente Validität: α = 0,0055 [0,05/9]).

#### Gewichtung der DiCoDi®-Subtests

Zusätzlich wurde eine ROC(„receiver operating characteristic“)-Analyse für alle DiCoDi®-Subtests durchgeführt. Die Fläche unter der ROC-Kurve („area under the curve“, AUC) diente der Klassifizierung der Testqualität. Die Klassifikation in Personen mit versus ohne kognitive Beeinträchtigung erfolgte entsprechend des kombinierten Index (siehe Methode) in der gemeinsamen Stichprobe aus KG und Personen mit Schwerhörigkeit (siehe Abb. 2). Basierend auf der ROC-Analyse und den Kriterien von Fengler et al. [22] wurden die Subtests gewichtet. Es wurden 1 Punkt bei einer AUC von 0,51–0,60, 2 Punkte bei 0,61–0,70, 3 bei 0,71–0,80, 4 bei 0,81–0,90 und 5 bei 0,91–1,00 vergeben. Das relative Gewicht ergab sich aus dem Anteil an der Punktsumme, wurde mit 50 multipliziert und auf ganze Zahlen gerundet. Durch die Rundung ergab sich ein maximaler Gesamtwert von 48 Punkten.

#### Alters- und Bildungskorrektur des DiCoDi®-Gesamtwerts

Die Alters- und Bildungskorrektur des DiCoDi®-Gesamtwertes erfolgte in der Stichprobe aus 174 Personen ohne kognitive Beeinträchtigung, die die DiCoDi®-Subtests vollständig bearbeiteten (siehe Abb. 2; Frauen: *n* = 99 [56,9 %]; Alter: *M* = 68,65 Jahre [11,0]; Bildung: *M* = 13,85 Jahre [2,99]). Aufgrund der Zusammenhänge mit Alter und Bildung wurde der DiCoDi®-Gesamtwert alters- und bildungskorrigiert. Die Gruppenaufteilung erfolgte demnach anhand der Kategorien Bildung (≥ 12 Jahre vs. < 12 Jahre) und Alter (> 65 Jahre vs. ≤ 65 Jahre). In der Altersgruppe bis 65 Jahre bestand keine signifikante Korrelation zwischen dem DiCoDi®-Gesamtwert und der Bildung (*p* > 0,05), weshalb eine Bildungskorrektur entfiel. Daraus resultieren 3 Gruppen:

- Alter > 65 Jahre, Bildung ≥ 12 Jahre (*n* = 73),
- Alter > 65 Jahre, Bildung < 12 Jahre (*n* = 33),
- Alter ≤ 65 Jahre (*n* = 68).

Die Gruppe „Alter ≤ 65 Jahre“ erzielte die besten Leistungen im DiCoDi®-Gesamtwert, weshalb sie als Referenzgruppe festgelegt wurde. Zur Angleichung von Alters- und Bildungseffekten erhielten die Gruppen „Alter > 65 Jahre und Bildung ≥ 12 Jahre“ und „Alter > 65 Jahre und Bildung < 12 Jahre“ auf Basis der deskriptiven Statistik der Referenzgruppe zusätzliche Punkte (siehe Tab. S4). Der maximal erreichbare Gesamtwert von 48 Punkten bleibt dabei unverändert.

#### Klassifikationsgenauigkeit

Um die Klassifikationsgenauigkeit des alters- und bildungskorrigierten DiCoDi®-Gesamtwerts zu bestimmen, wurde die AUC zwischen Personen mit und solchen ohne kognitive Beeinträchtigung für den DiCoDi®-Gesamtwert berechnet.

#### Schweregradeinteilung

Cut-off-Werte wurden auf Basis der deskriptiven Statistik der Personen ohne kognitive Beeinträchtigung (*n* = 174) für den DiCoDi®-Gesamtwert erstellt. Hierfür wurde der Mittelwert abzüglich 1,5 Standardabweichungen herangezogen. Daraus resultierten 2 Gruppen:

- keine Hinweise auf kognitive Beeinträchtigung (*n* = 160),
- Hinweise auf leichte bis mittlere kognitive Beeinträchtigung (*n* = 14).

Die deskriptive Statistik beider Gruppen in den Paper-and-pencil-Tests wurde berechnet.

#### Re-Test-Reliabilität

Die Re-Test-Reliabilität des DiCoDi® wurde mithilfe von 2‑seitigen Spearman-Rangkorrelationen in einer gesunden Stichprobe (*n* = 71) bestimmt.

## Ergebnisse

### Gruppenunterschiede: KG vs. Schwerhörigkeit

Tab. 2 präsentiert die deskriptive Statistik der KG und der Personen mit Schwerhörigkeit in den DiCoDi®-Subtests sowie die Ergebnisse der ANCOVA. Für die DiCoDi®-Subtests „Trail Making A“ und „Zeitgefühl“ ergab sich ein signifikanter Haupteffekt der Gruppe mit kleinen bis großen Effektstärken, wobei in allen anderen DiCoDi®-Subtests kein signifikanter Haupteffekt gefunden werden konnte (siehe Tab. 2). Insgesamt unterschieden sich die KG und die Personen mit Schwerhörigkeit in den meisten Subtests nicht signifikant und zeigten deskriptiv vergleichbare Leistungen (siehe Tab. 2).

**Tab. 2:** Deskriptive Statistik und Ergebnisse der Kovarianzanalysen („analysis of covariance“, *ANCOVA*) in den *DiCoDi®*(Digital Cognitive Diagnostics)-Subtests von Kontrollgruppe (*KG*) und Personen mit Schwerhörigkeit

	*M* (*SD*) [Range]		Ergebnisse der ANCOVA	
DiCoDi®-Subtest	KG (*n* = 200)	Personen mit Schwerhörigkeit (*n* = 173)	*F*(df, df), $\boldsymbol{_{\mathrm{p}} \upeta ^{2}}$	*p*-Wert
Wortpaare merken (max. 30 Punkte)	21,54 (7,0) [1,00–30,00]	22,17 (5,8) [5,00–30,00]	*F*(1, 368) = 6,20, $_{\mathrm{p}} \upeta ^{2}$ = 0,22	0,013
Wortpaare merken, verzögerter Abruf (max. 10 Punkte)	7,50 (2,7) [0,00–10,00]	7,85 (2,4) [1,00–10,00]	*F*(1, 368) = 7,56, $_{\mathrm{p}} \upeta ^{2}$ = 0,18	0,006
Trail Making A (≤ 300 s)	40,23 (28,3) [11,00–232,00]	33,87 (15,9) [16,00–147,00]	*F*(1, 369) = 21,52, $_{\mathrm{p}} \upeta ^{2}$ = 0,24	0,001*
Trail Making B (≤ 300 s)	70,21 (47,3) [21,00–300,00]	66,95 (43,2) [23,00–272,00]	*F*(1, 369) = 5,06, $_{\mathrm{p}} \upeta ^{2}$ = 0,19	0,025
Zahlen und Buchstaben sortieren (max. 15 Punkte)	11,16 (4,6) [2,00–15,00]	9,48 (5,7) [2,00–15,00]	*F*(1, 195) = 4,87, $_{\mathrm{p}} \upeta ^{2}$ = 0,16	0,029
Konzentration und Aufmerksamkeit (max. 20 Punkte)	15,31 (5,1) [0,00–20,00]	15,47 (5,1) [0,00–20,00]	*F*(1, 369) = 2,43, $_{\mathrm{p}} \upeta ^{2}$ = 0,08	0,120
Kreishüpfen (max. 27 Punkte)	10,94 (6,4) [0,00–27,00]	10,91 (6,8) [0,00–27,00]	*F*(1, 369) = 2,51, $_{\mathrm{p}} \upeta ^{2}$ = 0,16	0,114
Zeitgefühl (≤ 150 s)	54,94 (17,3) [6,00–113,00]	60,62 (20,2) [12,00–147,00]	*F*(1, 370) = 14,53, $_{\mathrm{p}} \upeta ^{2}$ = 0,05	0,001*
Farb-Figuren-Test (max. 10 Punkte)	5,10 (2,2) [0,00–10,00]	4,54 (2,1) [0,00–10,00]	*F*(1, 368) = 1,93, $_{\mathrm{p}} \upeta ^{2}$ = 0,09	0,166

*M* Mittelwert, *SD* „standard deviation“

*p*-Wert der ANCOVA mit Alter und Bildung als Kovariaten. Nach Bonferroni-Korrektur wurden *p*-Werte unter einem Signifikanzniveau von α = 0,0055 als signifikant gewertet

### Gruppenunterschiede: Personen mit vs. ohne kognitive Beeinträchtigung

Tab. 3 zeigt die deskriptive Statistik der Personen mit und ohne kognitive Beeinträchtigung in den DiCoDi®-Subtests sowie die ANCOVA-Ergebnisse. Für alle DiCoDi®-Subtests bis auf „Zahlen und Buchstaben sortieren“, „Zeitgefühl“ und „Farb-Figuren-Test“ ergab sich ein signifikanter Haupteffekt der Gruppe mit großen Effektstärken (siehe Tab. 3).

**Tab. 3:** Deskriptive Statistik und Ergebnisse der Kovarianzanalysen („analysis of covariance“, *ANCOVA*) in den *DiCoDi®*(Digital Cognitive Diagnostics)-Subtests der Personen mit und ohne kognitive Beeinträchtigung

	*M* (*SD*) [Range]		Ergebnisse der ANCOVA	
DiCoDi®-Subtest	Personen ohne kognitive Beeinträchtigung (*n* = 314)	Personen mit kognitiver Beeinträchtigung (*n* = 58)	*F*(df, df), $\boldsymbol{_{\mathrm{p}} \upeta ^{2}}$	*p*-Wert
Wortpaare merken (max. 30 Punkte)	22,78 (5,9) [4,00–30,00]	16,75 (7,2) [1,00–30,00]	*F*(1, 367) = 37,07, $_{\mathrm{p}} \upeta ^{2}$ = 0,28	0,001*
Wortpaare merken, verzögerter Abruf (max. 10 Punkte)	8,03 (2,3) [1,00–10,00]	5,68 (2,9) [0,00–10,00]	*F*(1, 367) = 35,44, $_{\mathrm{p}} \upeta ^{2}$ = 0,24	0,001*
Trail Making A (≤ 300 s)	34,23 (19,0) [11,00–208,00]	53,70 (36,4) [22,00–232,00]	*F*(1, 368) = 26,81, $_{\mathrm{p}} \upeta ^{2}$ = 0,25	0,001*
Trail Making B (≤ 300 s)	60,99 (36,6) [21,00–272,00]	107,69 (61,6) [36,00–300,00]	*F*(1, 368) = 51,77, $_{\mathrm{p}} \upeta ^{2}$ = 0,28	0,001*
Zahlen und Buchstaben sortieren (max. 15 Punkte)	10,97 (4,9) [2,00–15,00]	7,87 (5,1) [2,00–15,00]	*F*(1, 195) = 3,75, $_{\mathrm{p}} \upeta ^{2}$ = 0,16	0,054
Konzentration und Aufmerksamkeit (max. 20 Punkte)	15,99 (4,9) [0,00–20,00]	12,16 (5,2) [0,00–20,00]	*F*(1, 368) = 23,93, $_{\mathrm{p}} \upeta ^{2}$ = 0,13	0,001*
Kreishüpfen (max. 27 Punkte)	11,54 (6,4) [0,00–27,00]	7,83 (6,3) [0,00–27,00]	*F*(1, 368) = 10,27, $_{\mathrm{p}} \upeta ^{2}$ = 0,17	0,001*
Zeitgefühl (≤ 150 s)	57,81 (17,7) [6,00–122,00]	56,45 (24,6) [12,00–147,00]	*F*(1, 369) = 0,30, $_{\mathrm{p}} \upeta ^{2}$ = 0,02	0,863
Farb-Figuren Test (max. 10 Punkte)	5,10 (2,2) [0,00–10,00]	4,00 (1,9) [0,00–9,00]	*F*(1, 367) = 6,56, $_{\mathrm{p}} \upeta ^{2}$ = 0,10	0,011

*M* Mittelwert, *SD* „standard deviation“

*p*-Wert der ANCOVA mit Alter und Bildung als Kovariaten. Nach Bonferroni-Korrektur wurden *p*-Werte unter einem Signifikanzniveau von α = 0,0055 als signifikant gewertet. Zahlen und Buchstaben sortieren: *n* = 176 Personen ohne vs. *n* = 23 Personen mit kognitiver Beeinträchtigung

### Konvergente und divergente Validität

Alle DiCoDi®-Subtests zeigten signifikante, moderate bis starke Korrelationen mit den dazugehörigen Paper-and-pencil-Tests (Range *r*_s_ = 0,24–0,67; *p* <  0,000694; siehe Tab. S2). Zudem war kein DiCoDi®-Subtest signifikant mit dem BDI‑V korreliert (*p* > 0,05).

### Gewichtung der Subtests

In der ROC-Analyse wurden zur Bestimmung der AUC Personen mit und ohne kognitive Beeinträchtigung hinsichtlich der DiCoDi®-Subtests verglichen (siehe Tab. S3). Die Ergebnisse der ROC-Analyse deuten auf eine moderate bis gute Vorhersagegenauigkeit der DiCoDi®-Subtests hin (durchschnittliche AUC: 0,72). Auf Basis der ROC-Analyse und Fengler et al. [22] wurden die DiCoDi®-Subtests gewichtet (siehe Tab. S3).

### Alters- und Bildungskorrektur des DiCoDi®-Gesamtwerts

Der DiCoDi®-Gesamtwert korrelierte signifikant mit Alter (*p*  < 0,001) und Bildung (*p*  < 0,001), jedoch nicht mit Geschlecht (*p*  > 0,05), weshalb eine Alters- und eine Bildungskorrektur vorgenommen wurden (siehe Tab. S4). Nach der Korrektur erreichten die Personen der KG einen mittleren Gesamtwert von 44,65 (5,6) und die Personen mit Schwerhörigkeit einen mittleren Gesamtwert von 46,04 (4,1), wobei sich die Gruppen entsprechend dem Mann-Whitney-U-Test nicht signifikant (*p*  > 0,05) unterschieden.

### Klassifikationsgenauigkeit

Die AUC des alters- und bildungskorrigierten DiCoDi®-Gesamtwerts zwischen Personen mit und ohne kognitive Beeinträchtigung lag bei 0,80.

### Schweregradeinteilung

Basierend auf der deskriptiven Statistik der Personen ohne kognitive Beeinträchtigung im alters- und bildungskorrigierten DiCoDi®-Gesamtwert (*M* = 45,09 [5,2]) und unter Berücksichtigung von Augenscheinvalidität erfolgte eine Einteilung des DiCoDi®-Gesamtwerts (siehe Tab. 4). Die deskriptive Statistik der beiden Schweregradgruppen in den Paper-and-pencil-Tests kann der Tab. S5 entnommen werden.

**Tab. 4:** Schweregradeinteilung des *DiCoDi®*(Digital Cognitive Diagnostics)-Gesamtwerts

DiCoDi®-Gesamtwert	Interpretation	*n* (%)
38–48 Punkte	Keine Hinweise auf kognitive Beeinträchtigung	160 (92,0 %)
≤ 37 Punkte	Hinweise auf leichte bis mittlere kognitive Beeinträchtigung	14 (8,0 %)

### Re-Test-Reliabilität

Der DiCoDi®-Gesamtwert korreliert signifikant vom ersten (*M* = 44,97 [4,5]) zum zweiten Testzeitpunkt (*M* = 44,80 [4,4]), was auf eine gute Re-Test-Reliabilität hindeutet (*r*_s_ = 0,51; *p* = 0,005). Im Mittel lagen zwischen dem ersten und zweiten Testtermin 9,4 (7,1) Wochen.

## Diskussion

In der vorliegenden Studie wurde der DiCoDi® vorgestellt und evaluiert – ein tabletbasierter kognitiver Test für Menschen mit und ohne Schwerhörigkeit. Unsere Hauptergebnisse zeigen, dass

1. sich die KG und Personen mit Schwerhörigkeit größtenteils nicht im DiCoDi® unterscheiden, während sich Personen mit und ohne kognitive Beeinträchtigung deutlich unterscheiden;
2. Validität und Reliabilität hoch sind;
3. der DiCoDi® eine gute Klassifikationsgenauigkeit zeigt;
4. ein Cut-off-Wert von ≥ 38 im alters- und bildungskorrigierten Gesamtwert zur Erkennung kognitiver Defizite sinnvoll erscheint.

Die KG und Personen mit Schwerhörigkeit unterschieden sich im DiCoDi®-Gesamtwert und in den meisten Subtests nicht signifikant. Vergleichbare Ergebnisse berichteten auch Völter et al. [23] für den MoCA‑H. Allerdings erzielte die Gruppe mit Schwerhörigkeit in den DiCoDi®-Subtests „Trail Making A“ und „Zeitgefühl“ signifikant bessere Ergebnisse als die KG. Dieses Ergebnis steht im Kontrast zu bestehender Forschung; beispielsweise fanden Huber et al. [24] im TMT‑A keine Gruppenunterschiede. Möglicherweise können die durchschnittlich schlechteren Leistungen der KG im Vergleich zu Personen mit Schwerhörigkeit in der vorliegenden Studie durch eine deutlich höhere Varianz im Trail Making A (232 vs. 147 s) erklärt werden. Zudem ist Zeitgefühl eine relativ neue kognitive Aufgabe [25]. Meyers et al. [25] berichten Normdaten für eine 1‑min-Schätzung, wobei 53–68 s eine altersnormgerechte Leistung darstellen. In unserer Studie lagen beide Gruppen innerhalb dieses Bereichs. Studien zur kognitiven Schätzfähigkeit bei Menschen mit Schwerhörigkeit fehlen bislang.

Darüber hinaus erzielten Personen mit kognitiver Beeinträchtigung erwartungsgemäß schlechtere Leistungen in den DiCoDi®-Subtests als Personen ohne kognitive Beeinträchtigung – mit Ausnahme der Subtests „Zeitgefühl“, „Zahlen und Buchstaben sortieren“, und „Farb-Figuren-Test“. Bei letzteren beiden zeigte sich zwar kein signifikanter Unterschied, jedoch ein deskriptiver Trend in die erwartete Richtung. Eine mögliche Ursache des fehlenden Unterschieds im Subtest „Zahlen und Buchstaben sortieren“ könnte in der kleinen Stichprobengröße der Personen mit kognitiver Beeinträchtigung (*n* = 23) liegen. Möglicherweise sind Sprachverständnis (Farb-Figuren-Test) und kognitive Schätzfähigkeit (Zeitgefühl) erst bei ausgeprägteren kognitiven Beeinträchtigungen eingeschränkt [26, 27], weshalb in unserer Studie keine entsprechenden Unterschiede nachweisbar waren.

Neben einer hohen konvergenten und divergenten Validität zeigte der DiCoDi® eine hohe Re-Test-Reliabilität. Ähnliche Ergebnisse erzielten auch Völter et al. [23] im MoCA‑H und Lin et al. [7] im HI-MoCA. Mit einer AUC von 0,80 zeigt der DiCoDi®-Gesamtwert eine gute Klassifikationsgenauigkeit, jedoch erreicht beispielsweise der MoCA‑H eine Sensitivität und Spezifität von über 0,90 [8]. Es sollte jedoch betont werden, dass in dieser Studie keine formalen Diagnosen gestellt wurden. Vielmehr wurde untersucht, wie gut der DiCoDi® zwischen Personen mit und solchen ohne kognitive Beeinträchtigung differenzieren kann – basierend auf einem kombinierten Index aus 5 Kognitionstests. Ein ähnliches Vorgehen führt bei Kognitionstests für Menschen mit Schwerhörigkeit, wie dem O‑DEM und HI-MoCA, ebenfalls zu einer AUC von etwa 0,80 [10]. Ein nächster Schritt wäre die Validierung des DiCoDi® in einer Stichprobe mit formellen Diagnosen wie Demenz oder MCI („mild cognitive impairment“).

### Limitationen

Es fehlen objektive Hördaten für die KG, wodurch das Vorliegen einer Schwerhörigkeit nicht ausgeschlossen werden kann, da der Zusammenhang zwischen einer Schwerhörigkeit und deren subjektiver Wahrnehmung variieren kann [28]. Eine weitere Einschränkung liegt in der multizentrischen Datenerhebung, bei der die KG in Deutschland und die Personen mit Schwerhörigkeit in der Schweiz erhoben wurden. Zudem sorgt die Selektivität der Stichprobe der Personen mit Schwerhörigkeit für eine eingeschränkte Repräsentativität und Generalisierbarkeit der Ergebnisse. Auch die ungleiche Gruppenverteilung nach Schweregrad der Schwerhörigkeit stellt eine mögliche Limitation dar. Zudem umfasste die Stichprobe mit kognitiver Beeinträchtigung nur 58 Personen. Folgestudien sollten den DiCoDi® an einer größeren Kohorte tatsächlicher Patient*innen mit formaler Diagnose untersuchen. Aufgrund der geringen Stichprobengröße von Personen mit Hinweisen auf leichte bis mittlere kognitive Beeinträchtigung im DiCoDi® (*n* = 14) konnte bislang keine weitere Differenzierung des Schweregrads der kognitiven Beeinträchtigung mit dem DiCoDi® vorgenommen werden.

### Stärken

Die große Stichprobengröße ermöglicht eine hohe Power und eine höhere Generalisierbarkeit der Ergebnisse. Zudem können die digitale Durchführung und die automatische Auswertung des DiCoDi® Zeit- und Personalaufwand reduzieren. Außerdem ermöglicht der DiCoDi® innerhalb von 20 bis 30 min eine umfassende Überprüfung verschiedener kognitiver Funktionen. Dadurch bietet er eine differenziertere Erfassung kognitiver Leistungen als herkömmliche kognitive Screenings, ist dabei jedoch gleichzeitig hinreichend zeiteffizient und praktikabel, um im klinischen Alltag eingesetzt werden zu können. Der DiCoDi® stellt somit eine sehr kurze Testbatterie dar, die immer noch umfassend genug ist, um Therapieempfehlungen aus dem Testprofil abzuleiten. Darüber hinaus ist der DiCoDi® unabhängig von der Hörfähigkeit einsetzbar. So kann sichergestellt werden, dass schlechte Leistungen auf kognitive Einbußen zurückgehen und nicht auf mangelndes Instruktionsverständnis, falsche Item-Wahrnehmung oder erhöhte Ablenkung aufgrund einer geänderten Hörfähigkeit [6].

## Fazit für die Praxis

- Die Notwendigkeit eines schnellen und validen kognitiven Tests zur Früherkennung leichter bis mittlerer kognitiver Beeinträchtigungen bei Menschen mit Schwerhörigkeit wird angesichts der Zunahme von Demenzerkrankungen und Schwerhörigkeit deutlich.
- Bewährte kognitive Tests berücksichtigen auditive Einschränkungen unzureichend. Adaptionen für Menschen mit Schwerhörigkeit sind größtenteils noch Paper-and-pencil-basiert, was hohe zeitliche und personelle Kapazitäten erfordert.
- Der DiCoDi® (Digital Cognitive Diagnostics) ist ein einfach und digital durchführbarer Test, der durch die ausschließliche visuelle Stimuluspräsentation mögliche kognitive Defizite bei Menschen mit und ohne Schwerhörigkeit detektieren kann.
- Der DiCoDi® ersetzt keine neuropsychologische Testbatterie, liefert jedoch erste Hinweise auf mögliche kognitive Beeinträchtigungen.

## Supplementary Information

**Tab. S1** Übersicht der DiCoDi®-Subtests; **Tab. S2** Korrelationen zwischen den DiCoDi®-Subtests und den Paper-and-pencil-Tests (konvergente Validität); **Tab. S3** AUC-Werte der DiCoDi®-Subtests im Vergleich von Personen mit und ohne kognitive Beeinträchtigung; **Tab. S4** Alters- und Bildungskorrektur des DiCoDi®-Gesamtwerts; **Tab. S5 **Deskriptive Statistik der DiCoDi®-Schweregradgruppen in den Paper-and-pencil-Tests

## Data Availability

Die in dieser Studie erhobenen Datensätze können auf begründete Anfrage beim Korrespondenzautor angefordert werden.
