# Supplementary material for: Digital cognitive diagnostics (DiCoDi®): A tablet-based cognitive test for people with and without hearing loss
Source: HNO. 2026 Feb 2;74(10):664–72. [Article in German] doi: 10.1007/s00106-026-01722-8 (PMC13615026; doi:10.1007/s00106-026-01722-8)
Supplement: Supplementary file 1 [file 106_2026_1722_MOESM1_ESM.pdf]

Zusatzmaterial

DiCoDi® (Digital Cognitive Diagnostics):
Ein tablet-basierter kognitiver Test für Menschen mit und ohne Schwerhörigkeit

DiCoDi® (Digital Cognitive Diagnostics):
A tablet-based cognitive test for individuals with and without hearing loss

---

Tab. S1 Übersicht der DiCoDi®-Subtests ____ S. 2

Literatur zur Tab. S1 ____ S. 4

Tab. S2 Korrelationen zwischen den DiCoDi®-Subtests und dazugehörige Paper&Pencil-Tests (konvergente Validität) ____ S. 5

Tab. S3 AUC-Werte der DiCoDi®-Subtests im Vergleich von Personen mit und ohne kognitive Beeinträchtigung ____ S. 6

Tab. S4 Alters- und Bildungskorrektur des DiCoDi®-Gesamtwerts ____ S. 7

Tab. S5 Deskriptive Statistik der DiCoDi®-Schweregradgruppen in den Paper&Pencil-Tests ____ S. 8

Tab. S1 Übersicht der DiCoDi®-Subtests

DiCoDi®-Subtests	Kognitive Domäne/n	Aufgabe	Punktevergabe	Basierend auf
Wortpaare merken (3 Durchgänge)	Episodisch-verbales Gedächtnis (kurz- und mittelfristiges Gedächtnis), verbales Lernen	Zehn Wortpaare werden sukzessive dreimal dargeboten und jeweils unmittelbar abgefragt. Hierbei muss aus einem Wörterpool das richtige Wort angetippt werden	Für jedes richtig erinnerte Wort wird ein Punkt vergeben. Eine höhere Punktzahl entspricht einer besseren Leistung	Verbal Paired Associates (Wortpaare merken) der Wechsler Memory Scale-IV (WMS-IV; [1])
Wortpaare merken verzögerter Abruf	Episodisches Gedächtnis (mittelfristiges Gedächtnis)	Nach einer Verzögerungszeit von etwa 18 Minuten werden die Lern- und Behaltensleistung der Wortpaare erneut mithilfe eines „multiple choice“-Antwortformats abgefragt	Für jedes richtig erinnerte Wort wird ein Punkt vergeben. Eine höhere Punktzahl entspricht einer besseren Leistung	Verbal Paired Associates (Wortpaare merken) der Wechsler Memory Scale-IV (WMS-IV; [1])
Trail Making A	Kognitive Verarbeitungs-geschwindigkeit	Verstreute Zahlen von 1 bis 20 müssen in aufsteigender Reihenfolge so schnell wie möglich angetippt werden. Bei einem Tippfehler wird die falsche Zahl rot markiert. Erst nach Auswahl der korrekten Zahl werden beide Zahlen verbunden und die Aufgabe kann fortgesetzt werden	Es wird die Zeit in Sekunden gemessen. Die Anzahl der Fehler wird nicht separat erfasst, sondern die Fehler gehen indirekt in die Bearbeitungszeit ein. Eine längere Bearbeitungszeit entspricht einem schlechteren Ergebnis	Trail-Making-Test A (TMT-A; [2])
Trail Making B	Exekutive Funktionen in Form von kognitiver Flexibilität	Es müssen Zahlen und Buchstaben schnellstmöglich alternierend angetippt werden: Zahlen in aufsteigender, Buchstaben in alphabetischer Reihenfolge. Bei einem Tippfehler wird das falsche Item rot markiert. Erst nach Auswahl des korrekten Items werden die Buchstaben und Zahlen verbunden und die Aufgabe kann fortgesetzt werden	Es wird die Zeit in Sekunden gemessen. Die Anzahl der Fehler wird nicht separat erfasst, sondern die Fehler gehen indirekt in die Bearbeitungszeit ein. Eine längere Bearbeitungszeit entspricht einem schlechteren Ergebnis	Trail-Making-Test B (TMT-B; [2])

Zahlen und Buchstaben sortieren	Arbeitsgedächtnis	Unsortierte Buchstaben und Zahlenkombinationen müssen ihrer Reihenfolge nach geordnet werden. Die Reihenfolge besteht darin, zuerst die Zahlen aufsteigend zu sortieren, gefolgt von den Buchstaben in alphabetischer Ordnung (z. B. K, 5, A, 2 → 2, 5, A, K).	Wird der erste Versuch direkt korrekt gelöst, erhält die Testperson Punkte entsprechend der Länge der Buchstaben-Zahlen- Folge. Ist der erste Versuch nicht richtig, werden im zweiten Versuch Punkte vergeben: Bei einer komplett richtigen Antwort entspricht die Punktzahl der Anzahl der Items, bei einer teilweise richtigen Antwort der Anzahl der korrekten Items. Nach zwei falsch gelösten Versuchen derselben Sequenzlänge erfolgt der nächste Subtest. Eine höhere Punktzahl spiegelt eine bessere Leistung wider	Buchstaben-Zahlen-Folgen der Wechsler Adult Intelligence Scale (WAIS; [3])
Konzentration und Aufmerksamkeit	Kurzfristige Konzentration und Aufmerksamkeit	Auf dem Bildschirm werden Kreise präsentiert, die jeweils aus vier unterschiedlich eingefärbten Sektoren bestehen. Kreise mit den eingefärbten Sektoren entsprechend der zwei vorgegebenen Zielsymbole müssen innerhalb von 90 Sekunden angetippt werden	Pro Fehler (d. h. Auslassungen oder falsch Positive) wird ein Punkt von der Maximalpunktzahl (20 Punkte) abgezogen. Eine höhere Punktzahl entspricht einem besseren Ergebnis	Subtest Aufmerksamkeit der Aphasie-Check-Liste (ACL; [4])
Kreishüpfen	Visuospatiales Arbeitsgedächtnis	Es muss aus einem Pool mit neun Kreisen sukzessive eine vorgegebene Sequenz in umgekehrter Reihenfolge nachgetippt werden. Nachdem zweimal eine Sequenz derselben Länge falsch eingegeben wurde, wird zum nächsten Subtest übergegangen	Für jede korrekte Sequenz werden Punkte entsprechend ihrer Länge vergeben. Es werden keine Teilpunkte vergeben. Eine höhere Punktzahl bedeutet eine bessere Leistung	Corsi Block Tapping Task [5]
Zeitgefühl	Kognitives Schätzen (Beurteilung von Zeitintervallen), Exekutive	Es muss mit geschlossenen Augen eine Zeitspanne von 60 Sekun-	Je näher die Schätzung an den 60 Sekunden liegt, desto besser ist die Leistung	-

	Funktionen	den abgeschätzt werden		
Farb-Figuren Test	Sprachverständnis und Kurzzeitgedächtnis	Es werden unterschiedlich farbige und unterschiedlich große Symbole (Sonne, Mond und Sterne) auf dem Bildschirm dargeboten. Es müssen Instruktionen umgesetzt werden, wie z.B. „Tippen Sie entweder auf den kleinen roten Stern oder die kleine blaue Sonne.“	Je nach Schwierigkeitsgrad der Instruktion werden ein, zwei oder drei Punkte vergeben. Eine höhere Punktzahl deutet auf eine bessere Leistung hin	Farb-Figur-Test [4]

Literatur

1. Wechsler D (2009) The Wechsler memory scale - 4. Aufl. (WMS-IV). Pearson Assessments
2. Parkington J, Leiter R (1949) Partington’s Pathway Test. Psychological Service Center Journal 1:9–20
3. Wechsler D (1955) Manual for the Wechsler adult intelligence scale. The Psychological Corporation
4. Kalbe E, Reinhold N, Brand M et al (2005) A new test battery to assess aphasic disturbances and associated cognitive dysfunctions: German normative data on the Aphasia Check List. J Clin Exp Neuropsychol 27(7):779–794
5. Corsi (1972) Human memory and the medial temporal region of the brain. Dissertation McGill University

Tab. S2 Korrelationen zwischen den DiCoDi®-Subtests und Paper&Pencil-Tests (konvergente Validität)

	**DiCoDi®-Subtests**								
**Paper&Pencil - Tests (Validierung)**	Wort-paare merken	Wort-paare merken verzöger-ter Abruf	Trail Ma-king A	Trail Ma-king B	Zahlen und Buch-staben sortieren	Konzentra-tion und Aufmerk-sam-keit	Kreis-hüpfen	Zeit-gefühl	Farb-Figuren Test
Verbal Paired Associates (WMS-IV)	0,52***	0,46***	-0,31***	-0,40***	0,28**	0,36***	0,27***	0,05	0,33***
TMT-A Sek	-0,43***	-0,40***	0,65***	0,61***	-0,35***	-0,39***	-0,32***	-0,09	-0,28***
TMT-B Sek	-0,53***	-0,46***	0,60***	0,67***	-0,42***	-0,43***	-0,39***	-0,19*	-0,38***
Buchstaben-Zahlen-Folgen (WAIS)	0,38***	0,34***	-0,30***	-0,41***	0,29***	0,31***	0,29***	0,06	0,32***
Aufmerksam-keit (ACL)	0,32***	0,32***	-0,42***	-0,40***	0,19	0,24***	0,28***	0,18*	0,15
Corsi Block Tapping Task	0,35***	0,28***	-0,36***	-0,41***	0,24	0,32***	0,35***	0,10	0,30***
Farb-Figur-Test (ACL)	0,34***	0,33***	-0,26***	-0,29***	0,26*	0,31***	0,25***	0,01	0,29***

ACL = Aphasie-Check-Liste, Sek = Sekunden, TMT-A = Trail Making Test A, TMT-B = Trail Making Test B, WAIS = Wechsler Adult Intelligence Scale, WMS-IV = Wechsler Memory Scale-IV. Zweiseitige Spearman-Rang-korrelationen. Nach Bonferroni-Korrektur werden *p*-Werte unter einem Signifikanzniveau von α = 0,000694 als signifikant gewertet. **p* < 0,000694 ***p* < 0,000139 ****p* < 0,0000694.

Tab. S3 AUC-Werte der DiCoDi®-Subtests im Vergleich von Personen mit und ohne kognitive Beeinträchtigung

	Wortpaare merken	Wortpaare merken verzögerter Abruf	Trail Making A	Trail Making B	Zahlen und Buchstaben sortieren	Konzentration und Aufmerksamkeit	Kreishüpfen	Zeitgefühl	Farb-Figuren Test
AUC-Wert	0,78	0,81	0,75	0,79	0,67	0,76	0,71	0,56	0,64
Gewichtete Punkte	6	8	6	6	4	6	6	2	4

Tab. S4 Alters- und Bildungskorrektur des DiCoDi®-Gesamtwerts

Gruppe	*n*	*M* (*SD*) vor Korrektur	Punktekorrektur	*M* (*SD*) nach Korrektur
Alter ≤ 65 Jahre (Referenz-gruppe)	68	46,00 (2,7)	+ 0	46,00 (2,7)
Alter > 65 Jahre, Bildung ≥ 12 Jahre	73	40,63 (7,2)	+ 6	44,73 (5,9)
Alter > 65 Jahre, Bildung < 12 Jahre	33	36,42 (8,7)	+ 10	44,00 (6,9)

Tab. S5 Deskriptive Statistik der DiCoDi®-Schweregradgruppen in den Paper&Pencil-Tests

	M (*SD*)<br>[Range]	
Paper&Pencil-Tests	Keine Hinweise auf kognitive Be-einträchtigung<br>(*n*=160)	Hinweise auf leichte bis mittlere kognitive Beeinträchtigung<br>(*n*=14)
Verbal Paired Associates (WMS-IV; max. 24 Punkte)	18,22 (3,5)<br>[7,00-24,00]	13,64 (7,1)<br>[0,00-22,00]
TMT-A (≤ 300 Sek.)	37,94 (13,0)<br>[17,00-82,00]	48,43 (12,9)<br>[26,00-71,00]
TMT-B (≤ 300 Sek.)	84,35 (35,1)<br>[20,00-252,00]	129,51 (47,8)<br>[52,00-214,00]
Buchstaben-Zahlen-Folgen (WAIS; max. 21 Punkte)	10,86 (3,1)<br>[3,00-21,00]	8,79 (2,5)<br>[5,00-13,00]
Aufmerksamkeit (ACL; max 144 Punkte)	113,96 (32,6)<br>[0,00-144,00]	103,50 (22,2)<br>[63,00-136,00]
Corsi Block Tapping Task (max. 12 Punkte)	7,60 (2,0)<br>[1,00-12,00]	6,43 (2,1)<br>[2,00-10,00]
Farb-Figur-Test (ACL; max. 12 Punkte)	11,58 (0,7)<br>[8,00-12,00]	11,00 (0,6)<br>[10,00-12,00]

ACL = Aphasie-Check-Liste, Sek = Sekunden, TMT-A = Trail Making Test A, TMT-B = Trail Making Test B, WAIS = Wechsler Adult Intelligence Scale, WMS-IV = Wechsler Memory Scale-IV.
